# Is suicide risk 100-fold higher in people with HIV? A critical appraisal of a meta-analysis

**DOI:** 10.1136/gpsych-2025-102210

**Published:** 2025-08-14

**Authors:** Andreas D Haas, Yann Ruffieux, Mpho Tlali, Stephan Rabie, Matthias Egger

**Affiliations:** 1 Institute of Social and Preventive Medicine, University of Bern, Bern, Switzerland; 2 Centre for Integrated Data and Epidemiological Research, School of Public Health, University of Cape Town, Cape Town, South Africa; 3 HIV Mental Health Research Unit, Department of Psychiatry and Mental Health, University of Cape Town, Cape Town, South Africa; 4 Population Health Sciences, Bristol Medical School, University of Bristol, Bristol, UK; 5 Department of Infectious Diseases and Hospital Epidemiology, University Hospital Zurich, University of Zurich, Zurich, Switzerland

**Keywords:** Suicide, Meta-Analysis as Topic

**To the editor:**

“A recent systematic review shows that the risk of death by suicide is 100 times higher in people living with HIV than in the general population”—World Health Organization, 2022

The estimate that the risk of suicide is 100-fold higher in people with HIV (PWH) than in the general population is widely cited, including in the World Health Organization’s (WHO’s) World Mental Health report from which the above quote is taken.^1^ This figure originates from a systematic review and meta-analysis by Pelton and colleagues, who estimated the ‘lifetime incidence’ of suicide among PWH^2^ and directly compared this measure to the WHO’s annual global suicide mortality rate.

In this commentary, we argue that the estimate of a 100-fold increased suicide risk in PWH is based on a methodologically flawed comparison and is not supported by the data reported in the original studies. We begin by critically appraising Pelton and colleagues’ methodology. We then present our own meta-analysis, limited to studies deemed appropriate for comparing suicide incidence between PWH and the general population.

## Critical appraisal

### ‘Lifetime incidence’ is not a valid epidemiological measure of suicide incidence

Pelton *et al* calculated the ‘lifetime incidence’ of suicide by dividing the number of suicides by the sample size. In effect, they report the proportion of participants dying by suicide. For the remainder of this commentary, we will use the term ‘proportion dying by suicide’ to describe the data analysed by Pelton *et al*, rather than lifetime incidence. The proportion dying by suicide is not an appropriate measure for quantifying suicide incidence. Incidence is a measure of event occurrence.^3^ As noted in a foundational epidemiology textbook, ‘to measure the frequency of disease occurrence in a population, it is insufficient merely to record the number of people or the proportion of the population that is affected’ (p33).^3^ Instead, it is necessary to ‘account for the length of time each individual was in the population at risk for the event’.^3^ The epidemiological standard measure for quantifying the occurrence of events like suicide is the incidence rate, calculated as the number of events divided by the total person-time at risk (p34).^3^ Although reported in most original studies, incidence rates were not analysed by Pelton *et al.* Instead, they analysed the proportion dying by suicide.

### Inappropriate denominators for calculating suicide incidence

By definition, incidence rates require person-time data from the entire at-risk population, including individuals who did not experience the event of interest.^3^ Pelton *et al* included data from two cross-sectional studies^4 5^ that included only deceased PWH. They pooled these studies reporting the proportion dying by suicide among deceased PWH with studies that estimate the proportion dying by suicide among all individuals enrolled in HIV cohort studies—both deceased and alive—within the same meta-analysis. This approach is clearly inappropriate and undermines the validity of their pooled estimate.^6^

### Non-comparable metrics: comparing proportions and incidence rates

Pelton *et al* directly compared the pooled proportion dying by suicide with the global suicide rate reported by WHO. This comparison is fundamentally flawed. Pelton *et al*’s proportion dying by suicide reflects the proportion of participants enrolled in HIV cohort studies who died by suicide over multiyear follow-up without accounting for person-time at risk. It is not comparable to the WHO suicide rate, which is defined as the number of suicides occurring within a single calendar year divided by the population at risk during that year, thereby capturing suicide deaths per person-year at risk.^7^ This inappropriate comparison of different epidemiological measures—comparing an annual incidence rate to a proportion—substantially inflates the estimated suicide rate among PWH.

### Non-comparable case definitions: comparing suicide rates with composite mortality outcomes

Pelton *et al* pooled data from three studies that reported the incidence of composite outcomes combining suicide with other causes of death, such as accidental overdose.^8–10^ They then compared this pooled estimate, including non-suicide deaths, with WHO suicide mortality rates, which include only suicides (ie, deaths from intentional self-harm (International Classification of Diseases, ICD-10 codes X60–X84, Y87.0)) and exclude accidental deaths (Annex A, pp62–66 in WHO^11^). The use of inconsistent case definitions again substantially inflates the estimated relative suicide incidence among PWH.

### Inadequate reference population

Suicide rates vary widely across the countries included in the meta-analysis. For example, in 2021, suicide rates per 100 000 person-years ranged from 4.7 in Greece to 16.6 in France among the included countries.^7^ Rates also differ by sex. In the USA, for instance, the suicide rate in males was 24.7 per 100 000 compared with 6.5 per 100 000 in females. A meaningful comparison between PWH and the general population must be made using country-specific and sex-specific suicide rates. All the studies included in Pelton’s meta-analysis were conducted in Europe and North America, regions with suicide rates above the global average,^7^ and predominantly included male participants (ranging from 70% to 92%),^12–16^ who have substantially higher suicide rates than females.^7^ Comparing male-dominated PWH cohorts from high-income countries with global averages for both sexes, therefore, further inflates the estimated relative suicide incidence among PWH.

### Duplication of data

An additional concern was the duplication of data from two studies reporting on the same cohort: Ruffieux *et al* 16 and Keiser *et al*.^17^ Keiser *et al* should have been excluded from the meta-analysis to prevent duplication of data.

## Reanalysis: methods

To provide robust estimates of suicide risk among PWH, we reanalysed data from the studies included in Pelton *et al*’s meta-analysis^1^ that we deemed appropriate for estimating suicide incidence in PWH based on standard definitions. We compared the rates from these studies to country-specific and sex-specific suicide rates for the general population. The reanalysis followed four steps:

### Study selection

We selected studies included in Pelton *et al*’s meta-analysis^1^ that either reported suicide incidence rates based on the WHO classification of suicide^11^ or provided sufficient data—specifically the number of suicides and person-time at risk—to calculate them. Seven of the 12 studies met these criteria.^9 10 12–16^ Five studies were excluded for the following reasons: two included only deceased individuals instead of all PWH at risk of suicide,^4 5^ one reported incidence for a composite outcome that included non-suicide deaths,^8^ one lacked the necessary data to calculate incidence rates^18^ and one^17^ overlapped with another included study based on the same cohort.

### Data extraction

Three authors (ADH, MT and SR) independently extracted data on the first author, publication year, country, study period, study design, proportion of male participants, number of suicides among PWH, number of PWH at risk and total person-years of follow-up. Discrepancies were resolved through discussion.

### Estimation and meta-analysis of suicide incidence rates in PWH

We defined suicide according to ICD criteria as death due to intentional self-harm (ICD-10 codes X60–X84, Y87.0), excluding other causes of death like accidental poisoning (X40, X43, X46–X48, X49).^11^ Suicide incidence rates were calculated by dividing the number of suicide deaths by person-years at risk, and the results were expressed per 100 000 person-years. We then conducted a random-effects meta-analysis of incidence rates using the metafor package^19^ in R (V.4.4.2).

### Estimation and meta-analysis of suicide incidence ratios

We estimated standardised incidence ratios (SIRs) by comparing the suicide incidence rate in each study with the corresponding country-specific and sex-specific rates in the general population and then pooled the SIRs using meta-analysis. General population suicide rates were obtained from the WHO Global Health Observatory for each study’s country and mid-year, standardised to match the sex distribution of PWH in each study.^7^ For two studies,^9 13^ the sex distribution was not reported and therefore inferred from other sources.^20 21^ Since WHO data were only available from 2000 onward, we used the estimates from 2000 for studies with earlier mid-years. We then calculated SIRs by dividing the observed number of suicides among PWH by the expected number of suicides in the general population, assuming the sex distribution of the general population matched that of the PWH cohort. Finally, we conducted a random-effects meta-analysis of SIR using the metafor package.^19^

#### Sensitivity analysis and supplemental materials

In the sensitivity analysis, we excluded one study showing extremely high suicide rates in both HIV-positive and HIV-negative people who inject drugs.^10^ Further details on the analytic approach are provided in a technical appendix (see online supplemental file 1), with the data (see online supplemental file 2) and statistical code provided in a public repository.^22^

10.1136/gpsych-2025-102210.supp1 Supplementary data

10.1136/gpsych-2025-102210.supp2 Supplementary data

## Reanalysis: results

**Figure 1:**
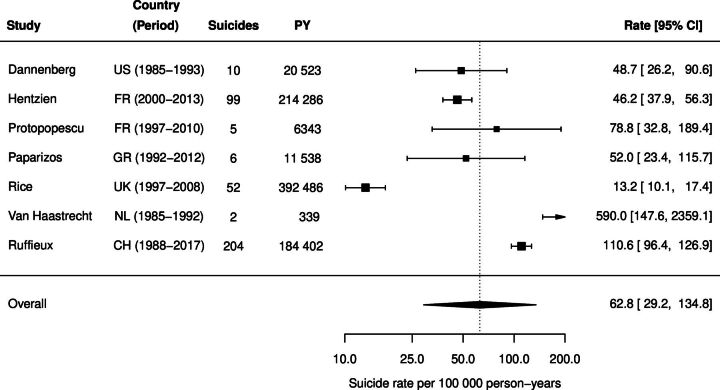
Suicide incidence rates among people with HIV in high-income countries. Forest plot of suicide incidence rates, per 100 000PY, with corresponding 95% CIs. Boxes represent study-specific incidence rates, with sizes proportional to study weights. Horizontal lines indicate 95% CIs. The diamond represents the pooled estimate. CH, Switzerland; CIs, confidence intervals; FR, France; GR, Greece; HIV, human immunodeficiency virus; NL, Netherlands; PY, person-years; UK, United Kingdom; US, United States.

Figure 1 presents the meta-analysis of suicide incidence rates in PWH from the seven included studies. Rates ranged from 13.2 suicides per 100 000 person-years in the UK (1997–2008) to 590.0 per 100 000 person-years in the Netherlands among injecting drug users in the era before effective HIV treatment became available (1985–1992). The pooled incidence rate across all studies was 62.8 per 100 000 person-years (95% confidence interval (CI) 29.2 to 134.8).

**Figure 2:**
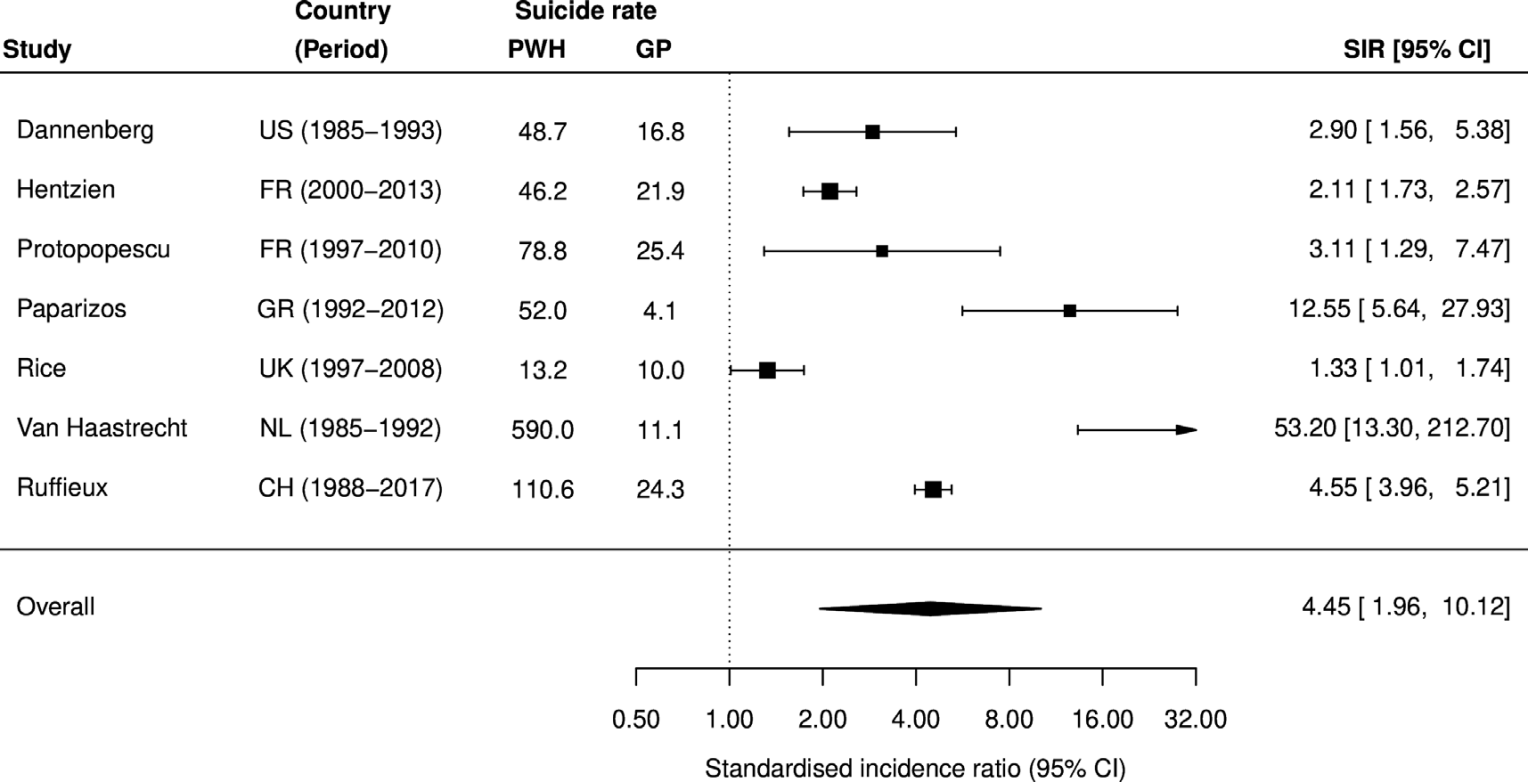
Standardised incidence ratios for suicide among people with HIV compared with the general population. Forest plot of SIRs and 95% CIs comparing suicide rates (per 100 000 PY) between PWH and GP of each study’s country. General population estimates are derived from WHO country-specific suicide rates^7^ for the mid-study year, weighted by the sex distribution of PWH in each study. Boxes represent study-specific SIRs, with sizes proportional to study weights. Horizontal lines indicate 95% CIs. The diamond represents the pooled estimate. CH, Switzerland; CIs, confidence intervals; FR, France; GP, general population; GR, Greece; HIV, human immunodeficiency virus; NL, Netherlands; PWH, people with HIV; PY, person-years; SIR, standardised incidence ratio; UK, United Kingdom; US, United States.

Figure 2 shows the SIRs comparing suicide risk among PWH to the general population. SIRs ranged from 1.33 in the UK to 53.20 in the Netherlands. The pooled SIR across all studies was 4.45 (95% CI 1.96 to 10.12).

In sensitivity analyses, excluding the Dutch study of people who inject drugs,^10^ the pooled suicide incidence rate was 47.8 per 100 000 person-years (95% CI 25.6 to 89.5), and the pooled SIR comparing PWH to the general population was 3.20 (95% CI 1.77 to 5.77).

## Discussion

PWH in Europe and North America have about four times higher suicide incidence compared with the general population, based on our reanalysis of seven studies included in Pelton *et al*’s meta-analysis. This estimate sharply contrasts with Pelton *et al*’s estimate of a 100-fold increase and illustrates that their result is not substantiated by the underlying studies. Pelton *et al*’s inflated estimate stems from a series of methodological errors, including the inappropriate comparison of proportions with incidence rates, combining deaths from suicide with other causes despite only suicide deaths being considered in the reference population, and the use of undifferentiated global average suicide rates as comparators for male-dominated high-income country cohorts.

Our SIR estimates align with primary studies comparing suicide rates in PWH and the general population. Dannenberg *et al* 12 reported an incidence rate ratio of 2.08 (95% CI 1.00 to 3.82) for suicide among HIV-positive military service applicants in the USA, adjusted for age, sex and race. Our corresponding SIR of 2.90 (95% CI 1.56 to 5.38) for this study is slightly higher, likely reflecting the absence of adjustment for age and race in our analysis. Similarly, our SIR of 4.55 (95% CI 3.96 to 5.21) for Switzerland is consistent with the age-SIR and sex-SIR reported in the original study.^16^

All studies included in our reanalysis were conducted in high-income countries in Europe or North America, where HIV disproportionately affects men who have sex with men and people who inject drugs—populations known to have elevated suicide risk independent of HIV status.^10 23–26^ The increased suicide rates observed among PWH in these studies may therefore reflect pre-existing vulnerabilities, rather than the effect of HIV itself. This interpretation is supported by the Dutch study, which reported similarly high suicide rates per 100 000 person-years among HIV-positive (590) and HIV-negative (530) people who inject drugs,^10^ indicating the excess risk is due to drug use rather than HIV.^10^ Gurm *et al*,^8^ included in Pelton’s meta-analysis, reported high rates for a composite of accidental poisoning or suicide—961 to 28 deaths per 100 000 person-years from 1998 to 2010—with 76% of deaths due to accidental poisoning (ICD-10 codes X40–X49), which WHO suicide rates exclude. We omitted this study from our reanalysis because it did not provide sufficient data to calculate suicide-specific rates.

Our study has several important limitations. First, we defined suicides according to the ICD criteria, aligning case definitions for PWH with those used by the WHO for the general population and excluding accidental deaths such as accidental overdose.^7 11^ Suicide rates in both PWH and the general population may be underestimated due to under-reporting and potential miscoding of suicides as accidental deaths.^27^ Second, the comparison of suicide rates between PWH and the general population does not account for potential confounding by age or other sociodemographic factors. Third, the estimated suicide rates among PWH are based on data from high-income countries and are not generalisable to high-prevalence settings with generalised HIV epidemics. In these contexts, where the majority of PWH reside, PWH tend to be more demographically representative of the general population and do not mainly include key populations at increased risk of suicide.^10 23–26^ As a result, the relative suicide risk among PWH in high-prevalence settings may be lower than that observed in this analysis.

In conclusion, PWH in Europe and North America have an approximately fourfold higher suicide incidence compared with the general population. The 100-fold increase reported by Pelton *et al* is not supported by the underlying studies. We trust the authors and journal editors will take appropriate action to maintain the integrity of the scientific record, following guidance from the Committee on Publication Ethics^28^ and the journal’s editorial policies.^29^

## Data Availability

Data, statistical code and a technical appendix detailing the analytic approach are provided as online supplemental materials to enable full replication of our analysis.
