# Supplementary material for: Is suicide risk 100-fold higher in people with HIV? A critical appraisal of a meta-analysis
Source: Gen Psychiatr. 2025 Aug 14;38(4):e102210. doi: 10.1136/gpsych-2025-102210 (PMC12359409; doi:10.1136/gpsych-2025-102210)
Supplement: Supplementary data [file gpsych-38-4-s001.pdf]

Technical Appendix: Meta-analysis of Suicide Incidence Rates and Rate Ratios

Data preparation and study selection

We extracted study-level data on suicides and person-years at risk among people with HIV (PWH). Suicide rates for the general population were obtained from the WHO Global Health Observatory for each study's country and mid-year, for males and females [1]. The mid-study year was calculated as the start year plus half the duration of the study period, rounded down to the nearest whole year.

Meta-analysis of incidence rates

We estimated study-specific suicide incidence rates per 100,000 person-years among PWH and pooled them using a random-effects meta-analysis. The primary outcome was the log incidence rate, calculated for each study as:

$$y_i = \log\left(\frac{e_i}{t_i}\right) \tag{1}$$

where e_i denotes the number of suicide events, and t_i the corresponding person-years of follow-up. Variances were computed using:

$$v_i = \frac{1}{e_i} \tag{2}$$

This transformation assumes a Poisson distribution for events, appropriate for rare outcomes such as suicide. We implement the random-effects model using restricted maximum likelihood (REML) estimation using the `metafor` package in R [2].

Comparison with general population: standardized incidence ratios (SIRs)

For each study i, we estimated the SIR, following the same formula used for standardized mortality ratios [3], as the ratio of observed to expected suicide deaths. The expected number was calculated under the counterfactual assumption that sex-specific suicide rates among PWH were equal to those in the general population of the study country during the midpoint of the study period. Specifically, we estimated:

$$\text{SIR} = \frac{e_i}{p_{\text{male},i} \cdot r_{\text{male},i} + p_{\text{female},i} \cdot r_{\text{female},i}} \tag{3}$$

Haas AD, et al. General Psychiatry 2025; 38:e102210. doi: 10.1136/gpsych-2025-102210

where e_i is the number of observed suicide deaths, $p_{\text{male},i}$ and $p_{\text{female},i}$ are the male and female person-years among PWH in study i, and $r_{\text{male},i}$ and $r_{\text{female},i}$ are the corresponding sex-specific suicide rates in the general population. Because the included studies did not report sex-specific person-years, we estimated these by distributing total person-years according to the proportion of male and female participants in each study.

Meta-analysis of standardized incidence ratios

We pooled the study-specific $\log(\text{SIR}_i)$s along with standard errors $1/\sqrt{e_i}$ [3] using a random-effects model via REML, as implemented in the `metafor` package [2].

References

[1] World Health Organization. Suicide Rates. 2025. https://www.who.int/data/gho/data/themes/mental-health/suicide-rates (accessed 4 April 2025)

[2] Viechtbauer W. Conducting Meta-Analyses in R with the metafor Package. *J Stat Softw.* 2010;36:1–48. doi:10.18637/jss.v036.i03

[3] Kirkwood, B. R., & Sterne, J. A. C. 2003. *Essential Medical Statistics* (2nd ed.). Blackwell Science.
